# Concomitant use of the novel extravascular implantable cardioverter-defibrillator with an epicardial pacemaker

**DOI:** 10.1016/j.hrcr.2025.07.009

**Published:** 2025-07-16

**Authors:** Vera Graup, Daniel Hofer, Alexander Breitenstein

**Affiliations:** Department of Cardiology, University Heart Center, University Hospital Zurich, Zurich, Switzerland

**Keywords:** Extravascular ICD, Substernal lead, Epicardial pacemaker, Combination of CIEDs, Device programming

**Key Teaching Points**

• The combination of an extravascular implantable cardioverter-defibrillator (EV-ICD) and a concomitant pacemaker should generally be avoided owing to the potential risk of device-device interaction. However, it may be clinically necessary in selected cases.
• Careful preprocedural planning is essential to minimize the risk of perioperative complications.
• Adjustment of standard device programming is crucial to reduce the risk of both under-and oversensing by the EV-ICD and the concomitant pacemaker.

## Introduction

The extravascular implantable cardioverter-defibrillator (EV-ICD) is a novel defibrillator with its lead placed substernally in the anterior mediastinum. Owing to limited pacing capabilities, patients requiring permanent stimulation are typically better suited for a transvenous defibrillator. However, in cases where this is not feasible (such as ongoing systemic infection), alternatives must be considered. We present a case where an EV-ICD was combined with an epicardial pacemaker to provide both defibrillation and pacing, while avoiding transvenous implantation.

## Case report

A 50-year-old man with a cardiomyopathy of unknown etiology was referred to our center for further evaluation 10 years ago. Despite an intense investigation including cardiac magnetic resonance imaging (MRI), positron emission tomography scan, and an endomyocardial biopsy, no underlying heart disease was identified. The patient refused genetic testing. His left ventricular ejection fraction was 45%–50%, and he was started on heart failure medication including bisoprolol, spironolactone, empagliflozin, and diuretics for fluid retention. In addition to his heart failure symptoms, he exhibited signs of sick sinus syndrome combined with a first-degree atrioventricular block on a 12-lead electrocardiogram. After extensive discussion with the patient, a transvenous dual-chamber pacemaker was implanted. Five years later, in 2020, he developed a sustained, hemodynamically relevant monomorphic ventricular tachycardia, prompting an upgrade to a transvenous dual-chamber implantable cardioverter-defibrillator (ICD) with extraction of the existing right ventricular lead, which was performed without complications. Unfortunately, 1 year later, he presented with a *Staphylococcus aureus*–associated device infection, likely owing to intravenous drug abuse. At that time, his cardiac condition had worsened with now persistent atrial fibrillation and complete atrioventricular block (whereas the left ventricular ejection fraction remained >50%). Owing to the risk of ongoing intravenous drug abuse, it was decided to fully extract the transvenous system and replace it with an epicardial dual-chamber ICD (Medtronic Evera MRI XT DR SureScan, Minneapolis, MN) with an intrapericardial shock coil (Medtronic 6937 52 cm) and an atrial (Medtronic 4968 35 cm) and right ventricular epicardial pace/sense lead (Greatbatch Medical 511212). The surgery was performed via a right-sided thoracotomy, and the patient was discharged uneventfully after 7 days. Three years later, a sudden increase in the high-voltage impedance suggestive of an ICD lead integrity failure was noted. Further evaluation confirmed a persistent shock lead issue, whereas the other electrical values were within normal limits, and hence, options to overcome this problem were discussed with the patient. A preinterventional subcutaneous ICD (S-ICD) electrocardiographic screening failed, and reverting to a transvenous device did not seem to be an option owing to the risk of ongoing drug abuse. Another thoracotomy with replacement of the intrapericardial shock lead was declined by the patient, and hence, the implantation of a substernal extravascular ICD (EV-ICD) combined with a downgrade of the epicardial system to a single-chamber pacemaker was recommended.

**Figure 1:**
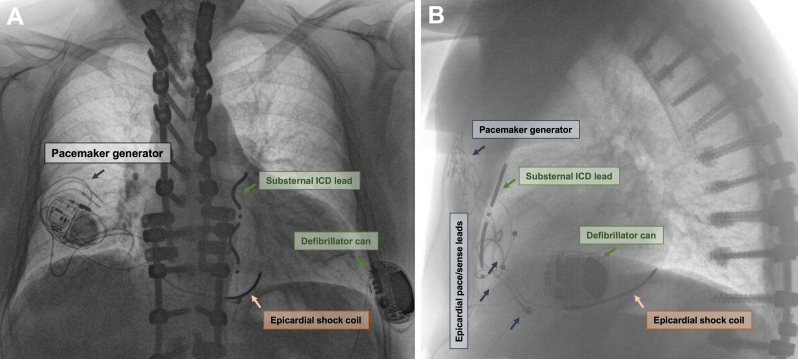
Final extravascular ICD device and lead position in antero-posterior (**A**) and lateral views (**B**). ICD = implantable cardioverter-defibrillator.

**Figure 2:**
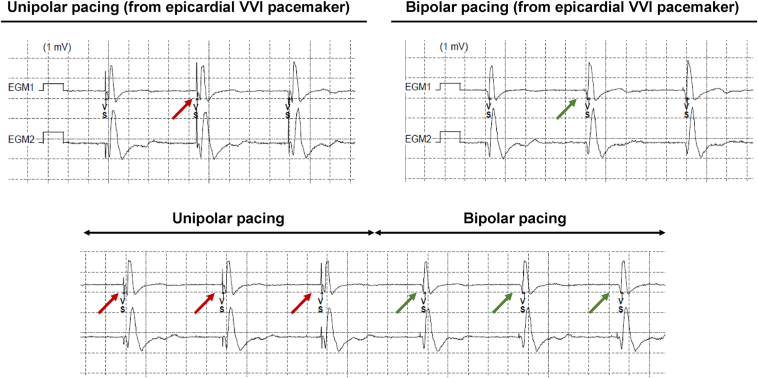
Bipolar sensing (Ring1-Ring2) via the extravascular implantable cardioverter-defibrillator during unipolar (left upper panel) and bipolar pacing (right upper panel) from the epicardial pacemaker. With changing from unipolar to bipolar pacing, the oversensing from the pacing artifact disappears (lower panel). EGM = electrogram.

The procedure was conducted under general anesthesia in a hybrid operating theater using fluoroscopic guidance. A preprocedural chest computed tomographic scan revealed enough distance between the posterior border of the sternum and the anterior cardiac structures. In addition, none of the epicardial leads were close to the area of access into the anterior mediastinum. During surgery, the antitachycardia function of the epicardial defibrillator was deactivated and the device reprogrammed with a low sensitivity along with a fixed pacing output of 3 V/0.4 ms in a bipolar configuration (similar to functional VOO mode). A horizontal subxiphoid incision was made at the standard location, allowing access to the mediastinum at the angulation between the xiphoid process and the left costal margin. Using the dedicated introducer tool, a 9F sheath was advanced substernally into the anterior mediastinum in maximal expiration without resistance despite previous right-sided thoracotomy (Supplemental Video 1). The lead was successfully positioned at the recommended anatomic location (Supplemental Video 2) with the tip just below the level of the tracheal bifurcation. Initial ventricular sensing, using a standard bipolar vector configuration (Ring1-Ring2), during ongoing bipolar ventricular pacing was excellent at 4.5 mV. However, minor atrial activity from the patient’s persistent atrial fibrillation was detected on the bipolar lead electrogram of the EV-ICD. To mitigate this, the lead was pulled caudally 1 cm, which eliminated the atrial fibrillation waves from the electrogram. Pacing at higher bipolar output levels (up to 8 V/0.4 ms) was sensed consistently via the EV-ICD lead, whereas pacing in a unipolar configuration resulted in oversensing of the pacing spike via the EV-ICD lead even at low output levels. The lead was secured to the fascia of the rectus muscle and connected to the generator in a previously prepared left lateral pocket. Subsequently, the epicardial ICD generator pocket was accessed, the shock coil and the atrial electrode were capped, and the ventricular lead was connected to a single-chamber pacemaker (Figure 1). Despite aggressive induction, a sustained ventricular arrhythmia could not be induced for testing of appropriate sensing and defibrillation efficacy of the substernal EV-ICD. As per institutional protocol for extravascular ICD implantation, all antitachycardia treatments remained deactivated until the following day. During the first device interrogation, the day after the procedure, the sensing performance of the defibrillator was assessed at different output levels and pacing configurations (bipolar vs unipolar) of the epicardial pacemaker (Figure 2). Oversensing of the pacing stimulus occurred at all output levels in unipolar configuration, whereas oversensing was not observed even at high outputs (8 V/0.4 ms) in bipolar configuration. The pacemaker was subsequently programmed to an output level of 3 V/0.4 ms in VVIR pacing mode with the capture management function disabled. The patient was discharged the next day with an uneventful follow-up (EV-ICD device programming in Supplemental Table 1).

## Discussion

The EV-ICD (Aurora EV-ICD, Medtronic) represents the latest advancement in the prevention of sudden arrhythmic death.^1^ Unlike conventional devices, the high-voltage lead in EV-ICD is positioned substernally between the posterior border of the sternum and the cardiac structures.^2^ In the EV-ICD, sensing occurs via 1 of 3 potential vectors (near field via Ring1-Ring2 or far field via Ring1/Ring2 and the generator) (Figure 2). The efficacy and safety of the EV-ICD were demonstrated in the EV-ICD pivotal investigational device exemption trial.^1^^,^^3^ Notably, no cases of mediastinitis or cardiac structural damage were observed among enrolled patients. Compared with the S-ICD,^4^ the EV-ICD offers additional capabilities including antitachycardia pacing and short-duration pause prevention pacing.^2^ A transvenous device is still favored in patients requiring chronic pacing or cardiac resynchronization therapy; however, certain clinical scenarios such as a persistent (nontreatable) high risk of recurrent device infection, intracardiac right-to-left shunt with a high risk of thromboembolic events, chronic occlusion, or congenital abnormalities of the superior venous system may preclude the implantation of a standard transvenous ICD in pacing-dependent patients.^5^, ^6^, ^7^ In such cases, alternative device strategies must be considered. A combination of an S-ICD with either a leadless^8^, ^9^, ^10^, ^11^, ^12^ or an epicardial pacemaker^13^, ^14^, ^15^ has been published including implantation and programming recommendations. When combining 2 devices, it is crucial to ensure a lack of interference on sensing and pacing and defibrillation function between them, and various aspects need to be taken into consideration, which is equally important for the EV-ICD.

### Preimplantation considerations

(1) Proximity between epicardial leads and EV-ICD electrode: given that the EV-ICD lead is near but not within the heart, sensing via the corresponding poles has a wider field than conventional transvenous devices. Hence, oversensing the pacing spike from the epicardial pacemaker is a significant risk and has to be avoided. In case of concomitant new implantation of an epicardial pacemaker, place the epicardial leads away from the anterior mediastinum (eg, choosing the left rather than the right atrial appendage for the atrial lead). In case of preexisting epicardial leads, plan to implant the substernal lead away from the epicardial leads (but still within the recommended borders for the EV-ICD implantation).
(2) Review the previous implantation report regarding the access site for the epicardial lead implantation (right vs left thoracotomy vs subxiphoidal access) and the incision site through the pericardium. Although an EV-ICD implantation after thoracotomy is usually possible,^16^ adhesions from a previous subxiphoidal access may complicate access into the anterior mediastinum.
(3) Review a preimplantation chest scan (either computed tomography or MRI) to delineate whether the course of the epicardial leads is in the proximity of the tunneling position of the EV-ICD lead.
(4) Review the “Power-on-Reset” and “End-of-Life” settings of the pacemaker and choose a model that avoids a unipolar pacing configuration if the device reverts into that pacing mode. Unipolar pacing remains a contraindication for the concomitant use of an EV-ICD.
(5) If pacing from the pacemaker is only possible in a unipolar mode, the implantation of an EV-ICD is contraindicated and other options should be discussed with the patient.

### Implantation considerations

(1) In patients with complete atrioventricular block and insufficient escape rhythm, program the pacemaker to a fixed rate in bipolar configuration and with a fixed output (eg, 3 V at 0.4 ms) with a low sensitivity (to avoid oversensing during cautery).
(2) In patients with sufficient intrinsic rhythm, program the pacemaker to a backup pacing mode (eg, heart rate of 30 beats/min) to allow an evaluation of EV-ICD sensing during intrinsic rhythm.
(3) Implant the EV-ICD lead not in the nearest proximity to the epicardial leads (but still within the recommended borders for EV-ICD implantation) under fluoroscopic guidance.
(4) For testing the accuracy of ventricular sensing:
  a. Sensing test during intrinsic activity (if present)
  b. Sensing test in a bipolar pacing configuration of the pacemaker with different output levels
  c. Sensing test in a unipolar pacing configuration of the pacemaker with different output levels
  d. Sensing test with the “Power-on-Reset” setting of the pacemaker
(5) Defibrillation testing of the Aurora EV-ICD:
  a. Induce ventricular fibrillation (VF) as per standard protocol.
  b. Verify appropriate sensing, detection, and treatment of the VF by the EV-ICD.
  c. Confirm appropriate sensing and detection of VF by the epicardial pacemaker to avoid ventricular undersensing and inappropriate pacing during VF.

### Postimplantation considerations

(1) Perform sensing test again in bipolar and unipolar pacing configurations of the epicardial pacemaker at different output levels.
(2) Program the epicardial pacemaker in a bipolar pacing configuration.
(3) Deactivate the capture management function of the epicardial pacemaker or program it to “Monitor only” to prevent potential pacing artifact oversensing at increased output levels.
(4) EV-ICD after shock pacing and pause prevention pacing: program off
(5) EV-ICD Wavelet autocollection:
  a. Non–pacemaker-dependent patients: program off and only manually collect a template during intrinsic rhythm. Check the Wavelet template at every follow-up.
  b. Pacemaker-dependent patients: turn Wavelet permanently off.
  To the best of our knowledge, this is the first published case of a concomitant use of an EV-ICD implantation and an epicardial pacemaker. The complex interaction between both devices requires careful testing of the devices, programming, and follow-up by a specialized center.

## Conclusion

Concomitant use of the EV-ICD and epicardial pacemakers is not recommended by the manufacturer at this time, but cannot be completely avoided depending on the clinical situation. If these devices are used in the same patient, careful implantation and programming considerations are necessary to overcome potential interactions between the 2 devices.

## Disclosures

V.G. has no conflict of interest. D.H. reports educational grants, consultant or speaker fees, and fellowship support from Abbott, Medtronic, Biotronik, Boston Scientific, Biosense Webster, Novartis, Bayer, Pfizer, and Spectranetics. A.B. has received consulting/presenter fees from Abbott, AngioDynamics, Bayer Health Care, Biosense Webster, Biotronik, Bristol Myers Squibb/Pfizer, Boston Scientific, Cook Medical, Daiichi Sankyo, Medtronic, and Philips.
