# Supplementary material for: Concomitant use of the novel extravascular implantable cardioverter-defibrillator with an epicardial pacemaker
Source: HeartRhythm Case Rep. 2025 Jul 16;11(10):1018–21. doi: 10.1016/j.hrcr.2025.07.009 (PMC12666895; doi:10.1016/j.hrcr.2025.07.009)
Supplement: Supplemental Table [file mmc1.docx]

**Supplemental table 1. EV-ICD device programming.**

Sensing Ring1-Ring2, RV sensitivity 0.150 mV, oversensing prevention 4

VF zone Active HR > 200 bpm 30/40 40J x6

FVT zone Active HR 200-240 bpm 30/40 3x Burst, 40J x5

VT zone OFF

VT monitor ON 176 bpm 32

Bradycardia OFF

Enhancements ON : VT monitor, High Rate Timeout ON, T Wave, Noise (Timeout)
