# Supplementary material for: Concomitant use of the novel extravascular implantable cardioverter-defibrillator with an epicardial pacemaker
Source: HeartRhythm Case Rep. 2025 Jul 16;11(10):1018–21. doi: 10.1016/j.hrcr.2025.07.009 (PMC12666895; doi:10.1016/j.hrcr.2025.07.009)
Supplement: Supplementary Material [file mmc2.docx]

**Legends supplementary videos**

Video 1. Advancing the tunnelling rod in lateral view.

Video 2. Final lead position in anterior-posterior view.

**Legend supplementary table 1**

EV-ICD device programming.
